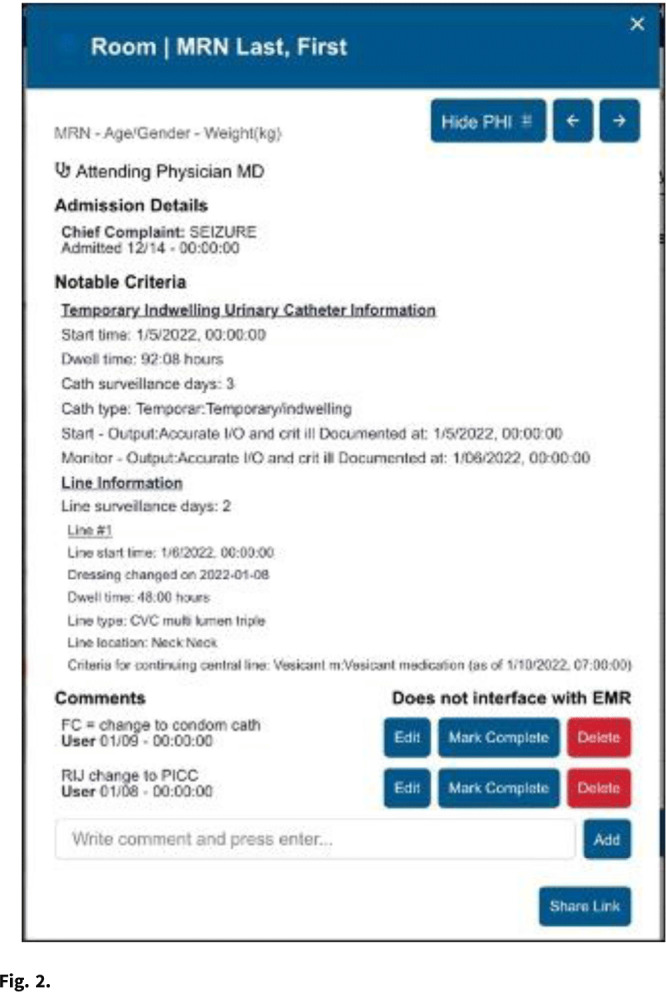# Creation of data application to facilitate device-monitoring safety for infection prevention

**DOI:** 10.1017/ash.2022.210

**Published:** 2022-05-16

**Authors:** Julia Moody, Quint Robinson, Trevor Townsend, Tyler Forehand, E. Jackie Blanchard, Kenneth Sands

## Abstract

**Background:** Efficient monitoring of devices to ensure timely removal is an ongoing challenge. There is a need for data visualization products that can aggregate disparate data streams to support device reviews, increase consistency across changes in caregiver teams, and synergize with people and operational processes within and across regional acute-care facilities. **Methods:** A data display application was developed to provide data from nearly any source in a consistent visual representation that could be used in real time. The infection prevention (IP) overlay combined data related to urinary catheters, central vascular catheters, and femoral vascular catheters from the electronic health record system. Clinical and data experts collaborated to develop data definitions, inclusion criteria, and report components. The application display indicated the current catheter or device status of each patient facility-wide, organized by service unit (Fig. 1). Additional patient information could be accessed from within the application, and a comment feature allowed caregivers to communicate directly through the tool (Fig. 2). **Results:** Pilot implementation began February 2021, and the NATE IP application was live for all users (unit and facility leaders, providers, infection preventionists, etc) as of July 2021. The tool is currently available for use at 171 acute-care hospitals within the HCA healthcare system, and it accommodated 3 different electronic medical record systems. Usage peaked in August 2021, with an average of 1,700 views per day. Daily utilization maximum ranges from 1,100 to 1,500 views per day, with an average of ~1,300 views per day. The tool is used during daily patient safety rounds, including weekends and holidays. User feedback was overwhelmingly positive, with users reporting an increase in communication, streamlined documentation, improved tracking of reasons to retain, and increased accountability for daily updates. During the proof-of-concept implementation, zero bugs were identified and several feature enhancements were implemented, including addition of port status and device-day reporting counts. Planned enhancements include mupirocin and chlorhexidine bathing use, isolation precaution use, and blood cultures ordered >3 days after admission. **Conclusions:** The NATE IP tool brings together data related devices into a single view for use by direct caregivers and all levels of leadership. Development of this or similar tools to consolidate various data streams into a central tool facilitates improved communication and consistency between caregiver teams. It also drives operational efficiencies and improves safety. Expansion to incorporate notifications related to potential issue will expand the proactive utility of this tool.

**Funding:** None

**Disclosures:** None

**Figure:**
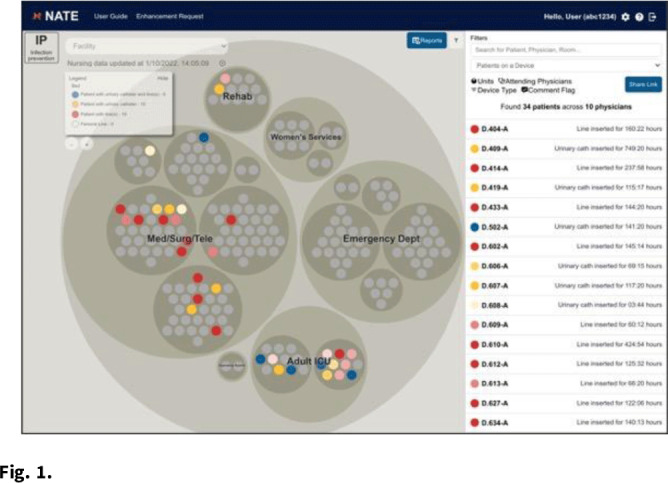

**Figure:**